# Gestational Diabetes Mellitus: Screening and Outcomes in Southern Italian Pregnant Women

**DOI:** 10.1155/2013/387495

**Published:** 2013-09-05

**Authors:** Carmelo Capula, Eusebio Chiefari, Anna Vero, Biagio Arcidiacono, Stefania Iiritano, Luigi Puccio, Vittorio Pullano, Daniela P. Foti, Antonio Brunetti, Raffaella Vero

**Affiliations:** ^1^Complex Operative Structure Endocrinology-Diabetology, Pugliese-Ciaccio Hospital, 88100 Catanzaro, Italy; ^2^Department of Health Sciences, University “Magna Græcia” of Catanzaro, Viale Europa (Località Germaneto), 88100 Catanzaro, Italy

## Abstract

Recent Italian guidelines exclude women <35 years old, without risk factors for gestational diabetes mellitus (GDM), from screening for GDM. To determine the effectiveness of these measures with respect to the International Association of the Diabetes and Pregnancy Study Groups (IADPSG) criteria, we evaluated 2,448 pregnant women retrospectively enrolled in Calabria, southern Italy. GDM was diagnosed following the IADPSG 2010 criteria. Among 538 women <35 years old, without risk factors, who would have not been tested according to the Italian guidelines, we diagnosed GDM in 171 (31.8%) pregnants (7.0% of total pregnants). Diagnosis was made at baseline (55.6%), 1 hour (39.8%), or 2 hours (4.7%) during OGTT. Despite of appropriate treatment, GDM represented a risk factor for cesarean section, polyhydramnios, increased birth weight, admission to neonatal intensive care units, and large for gestational age. These outcomes were similar to those observed in GDM women at high risk for GDM. In conclusion, Italian recommendations failed to identify 7.0% of women with GDM, when compared to IADPSG criteria. The risk for adverse hyperglycaemic-related outcomes is similar in low-risk and high-risk pregnants with GDM. To limit costs of GDM screening, our data suggest to restrict OGTT to two steps (baseline and 1 hour).

## 1. Introduction

Gestational diabetes mellitus (GDM) is defined as any degree of glucose intolerance with onset or first recognition during pregnancy [1]. Incidence of GDM is increasing worldwide for recent trends in obesity and advancing maternal age, with huge healthcare and economic costs [2, 3]. Women exposed to GDM are at high risk for pregnancy complications [4–6], future type 2 diabetes mellitus (DM), and cardiovascular disease [7–9]. In particular, several lines of evidence indicate a continuum of risk for adverse pregnancy outcomes for mothers and their offsprings related to increasing maternal glucose levels [10, 11], whereas treatment to reduce maternal glucose levels reduces this risk [12–14]. Based on these evidences, to identify women at risk for adverse pregnancy outcomes and improve prognosis through evidence-based interventions, recent tight diagnostic criteria for GDM have been introduced by the International Association of the Diabetes and Pregnancy Study Groups (IADPSG) [15]. Diagnosis requires a 75 g oral glucose tolerance test (OGTT) carried out between 24 and 28 weeks of gestation in all women not previously found to have overt diabetes or GDM, considering glycaemia of 92 mg/dL at baseline, 180 mg/dL at 1 h, and 153 mg/dL at 2 h from glucose load as cut-offs. To diagnose GDM, it is sufficient that only one of these thresholds is equaled or exceeded [15].

As expected, IADPSG criteria drastically increased the number of GDM diagnosis, with respect to most of the previously adopted criteria [16–20], posing a challenge to healthcare systems. In addition, many authors raised several doubts about the IADPSG criteria and their cost benefit impact [21–26], suggesting a major caution in worldwide adoption of these criteria, without further evidence. On the other hand, imposing universal strategies and standards appears little practicable, given the ethnic and regional variation in GDM prevalence and the different resources available. As a paradigm of this international controversy, the American Diabetes Association (ADA) has adopted the IADPSG recommendations since December 2010, while the American College of Obstetricians and Gynecologists (ACOG) still adopts previous criteria, including the two-step screening strategy [27, 28].

In an attempt to reduce maternal and neonatal outcomes, and avoid implications of clinical and economic overdiagnosis, linked to IADPSG criteria, in July 2011 an expert consensus supported by the Italian Ministry of Health introduced new guidelines for GDM screening (http://www.salute.gov.it/imgs/C_17_pubblicazioni_1436_allegato.pdf). Following these criteria, OGTT in Italy must be performed in all pregnants ≥35 years old or in women <35 years old in the presence of risk factors for GDM, including body mass index (BMI) ≥ 25, previous GDM, previous newborn with macrosomy, family history of type 2 DM, and ethnicity at high risk.

Thus, our aim was to verify—in an Italian population—the effectiveness of the new guidelines, with respect to the IADPSG criteria, in preventing maternal and neonatal outcomes in women <35 years old without risk factors for GDM.

## 2. Methods

### 2.1. Study Population

This is a retrospective population-based study involving 2,448 Caucasian pregnant women attending the Complex Operative Structure of Endocrinology-Diabetology, Pugliese-Ciaccio Hospital, Catanzaro, Calabria, Italy, from May 2010 to December 2012 for GDM screening and delivering in the same hospital. Calabrian population is characterized by a higher prevalence of GDM, type 2 DM, and obesity, when compared to the entire Italian population (http://www.istat.it). In particular, GDM diagnosis has doubled (from 13% to 28%) after the introduction of IADPSG criteria [29].

All pregnant women underwent OGTT screening between 24 and 28 weeks of gestation, including those <35 years old without risk factors for GDM, for whom, on the basis of the last Italian Ministry of Health guidelines, OGTT is not indicated. Gestational age was confirmed by ultrasonography examination. In all cases, diagnosis of GDM was made in accordance with the IADPSG guidelines [15]. All consecutive pregnant women with GDM were included in the study. Women with preexisting type 1 or type 2 DM, as defined by ADA criteria [1], with active chronic systemic disease, and with multifetal gestation were excluded.

In all women, blood samples were obtained after at least 8 hours of overnight fast and one and two hours after 75 g oral glucose load [1]. Women with GDM received individualized diet and/or insulin treatment, performed self-monitoring of their blood glucose (fasting, and 1 hr after breakfast, lunch, and dinner) daily with a portable glucometer, and underwent periodical clinical and biochemical evaluations (every 2 weeks or more frequently when appropriate). The goals of treatment were those indicated by ADA recommendations [30].

Anamnestic, clinical, and biochemical parameters were assessed for all women. Anamnestic information included age, parity, ethnicity, family history of diabetes (first- or second-degree relatives), and self-reported prepregnancy weight. The study was approved by the local ethics committee and a written consent was obtained from all women.

### 2.2. Study Outcomes

Outcomes among pregnant women included the mode of birth (cesarean delivery and labor induction), preterm delivery (delivery before 37 weeks of gestation), gestational hypertension, preeclampsia, polyhydramnios, and oligohydramnios. Gestational hypertension was defined as a blood pressure of at least 140/90 mmHg on two occasions at least 4 hr apart, or one elevated blood pressure value subsequently treated with medication. Preeclampsia was defined as an elevation in blood pressure together with proteinuria (≥300 mg/24 hr), or with either elevated liver enzyme levels (aspartate aminotransferase level ≥70 U per liter) or thrombocytopenia (platelet count < 100,000 per cubic millimeter).

Outcomes among the infants included death, stillbirth, dystocia (clinically defined), bone fracture, nerve palsy, admission to the neonatal intensive care unit (NICU), respiratory complications, such as distress syndrome (RDS, defined by the need for supplemental oxygen in the neonatal nursery beyond four hr after birth) and transient tachypnea of newborn (TTN), increased birth weight, macrosomy (defined as birth weight greater than 4000 g) [31], large size for gestational age (LGA, defined by a birth weight exceeding the 90th percentile on standard charts) [31], small size for gestational age (SGA, birth weight below the 10th percentile) [31], and metabolic complications (including hypocalcemia, hemoglobin level ≥ 20 g/dL, hypoglycemia, and phototherapy-requiring hyperbilirubinemia). Blood sample for fasting glucose determination was collected within 2 hr after birth. Neonatal hypoglycemia was defined as a value less than 35 mg/dL [32]. Serum bilirubin was evaluated between 16 and 36 hr after birth. A value greater than the 95th percentile for any given point after birth was considered to be elevated.

### 2.3. Statistical Analysis

Continuous variables are expressed as median and mean ± standard deviation, and categorical variables as numbers and percentages. The nonparametric Mann-Whitney test was used for comparisons of continuous variables and the 2-tailed Fisher exact test was used for comparisons of proportions. Generally, a significance level of 0.05 was set for a type I error in all analyses. GDM and other maternal features were analyzed as either dichotomous traits or continuous quantitative traits using regression models. Logistic regression analysis was used to evaluate the effect of GDM as possible risk factor for maternal and neonatal outcomes, adding appropriate covariates. Odds ratios (OR) with 95% confidence bounds were calculated. Linear regression analysis was employed to compare quantitative maternal traits at enrollment with respect to outcomes between two groups after adjustment for covariates. Each quantitative trait was tested for normality using the Shapiro-Wilk normality test, and, when required, it was log transformed. Furthermore, Pearson correlation coefficients were calculated to assess the strength of correlations between prepregnancy BMI and dystocia and the various outcomes independently from GDM. Data were analyzed with SPSS 20.0 software (SPSS Inc., Chicago, IL, USA). Post hoc statistical power calculations were performed with G*Power 3.1.3 software (Franz Faul, Kiel University, Kiel, Germany).

## 3. Results

### 3.1. Prevalence of GDM

**Figure 1:**
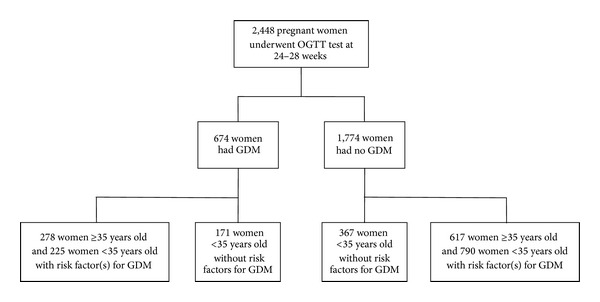
Enrollment of pregnant women.

Of the 2,448 pregnants screened from May 2010 to December 2012, 674 (27.5%) women were diagnosed as affected by GDM (Figure 1). Overall, 538 pregnant women <35 years old without any risk factor for GDM were screened; among these, 171 (31.8%) had GDM, and 367 (68.2%) had no GDM (Figure 1). Thus, following the recent Italian recommendations, these 171 women should have not been screened, and, therefore, they would have not been diagnosed.  Table 1(a) summarizes the demographic, anthropometric, clinical, and biochemical characteristics of both these cohorts of women. Maternal age, pregravidic BMI, and both increasing weight at OGTT time and at delivery were significantly higher (*P* < 0.001) in GDM groups, in addition to glycaemic levels after fast and glucose load (Table 1(a)). Out of 171 GDM women, 47 (27.5%) received insulin, whereas the remaining 124 (72.5%) were treated with appropriate diet. GDM treatment appeared efficacious, as indicated by a significant reduction of HbA1c at delivery, with respect to HbA1c at OGTT time (5.1% ± 0.3 versus 5.2 ± 0.3, *P* = 0.011), and as shown by the control of increasing weight at delivery with respect to OGTT time (10.3 kg ± 3.4 versus 9.8 ± 3.4, *P* = 0.131) (Table 1(b)). It was notable that diagnosis of GDM was made for 95 women (55.5%) at baseline, for 68 (39.8%) at 1 hr, and for 8 (4.7%) at 2 hr during OGTT. The only adverse event in these 8 women was a case of polyhydramnios.

### 3.2. Maternal and Neonatal Outcomes in Women <35 Years without Risk Factors

To verify whether the diagnosis of GDM in women <35 years without risk factors had effect on maternal and neonatal adverse events, despite of adequate glycaemic control (Table 1(b)), we performed a logistic regression analysis, in which GDM was the dependent variable.

The rate of primary cesarean section resulted significantly more common in the GDM group than in the control group (29.8 percent versus 15.3 percent; odds ratio 2.36, 95% confidence interval (CI) 1.53–3.64, *P* < 0.001). After correction for age, pregravidic BMI, and parity, association between GDM and primary cesarean section remained significant (adjusted odds ratio (AOR) 2.07, 95% CI 1.27–3.37, *P* = 0.003) (Table 2(a)). Secondary cesarean section in women that already had a previous cesarean delivery (AOR 5.05, 95% CI 2.11–12.08, *P* < 0.001) was strongly associated with GDM, whereas cesarean section after vaginal labor and labor induction were similar in the two groups (adjusted *P* = 0.401 and adjusted *P* = 0.177, resp.) (Table 2(a)). The sum of gestational hypertension and preeclampsia was associated with GDM (OR 2.44, 95% CI 1.05–5.65, *P* = 0.037), but this association missed its significance after adjustment for age, pregravidic BMI, and parity (AOR 2.03, 95% CI 0.83–4.97; *P* = 0.120) (Table 2(a)), similar to preterm delivery (OR 2.43, 95% CI 1.11–5.29, *P* = 0.025 and AOR 1.65, 95% CI 0.32–8.51, *P* = 0.549). Instead, a diagnosis of GDM was associated with polyhydramnios, even after correction for age, pregravidic BMI, and parity (AOR 4.48, 95% CI, 1.20–16.73, *P* = 0.025) (Table 2(a)). No association was observed with fetal distress and oligohydramnios, even if their rates in GDM group were higher when compared with the healthy group (Table 2(a)).

The newborns of GDM women showed a significant higher weight (*P* < 0.001), after correction for maternal age, pregravidic BMI, and gestational age at birth. No stillbirth, neonatal deaths, or nerve palsy occurred among the infants of mothers in both groups. No significant differences for other serious perinatal outcomes among the newborns (including shoulder dystocia and bone fracture) were observed (AOR 1.46, 95% CI 0.81–2.63, *P* = 0.202) (Table 2(b)). Instead, diagnosis of GDM was strongly associated with neonatal admission to NICU, also after adjustment for age, BMI, parity, and neonatal weight (AOR 4.39, 95% CI 1.44–13.37, *P* = 0.009) (Table 2(b)). Neonatal respiratory problems at delivery, including RDS and TTN, were not statistically different between the two groups (Table 2(b)). Furthermore, no difference was observed for macrosomy, whereas significantly more infants in the GDM group were LGA (AOR 3.53, 95% CI 1.34–9.34, *P* = 0.011) (Table 2(b)). There was no significant difference between groups in the proportion of infants who were SGA. GDM appeared associated with metabolic complications (OR 2.86, 95% CI 1.05–7.80, *P* = 0.041), but this association was missed after correction for age, BMI, parity, and gestational age at birth (Table 2(b)).

All significant associations were independent from BMI. However, we verified by Pearson's test that prepregnancy BMI was correlated with primary cesarean section (0.103, *P* = 0.017), neonatal weight (0.122, *P* = 0.005), and LGA (0.113, *P* = 0.009) independently from GDM. No association was observed with other outcomes.

### 3.3. Comparison among GDM Women

We compared proportions of maternal and neonatal outcomes in <35 years old GDM women without any risk factor for GDM with proportions of the same outcomes in GDM women ≥35 years old or with risk factor(s) for GDM (*N* = 503) (Figure 1). As reported in Tables 3(a) and 3(b), no significant differences were observed among the two Calabrian groups of GDM women, except for a higher rate of primary cesarean section in the first group (29.8 versus 15.9, *P* < 0.001) that was explained by a higher prevalence of breech presentation (10.5% versus 5.0%, *P* = 0.017) and nulliparity (56.1% versus 32.8%, *P* < 0.001), and a lower rate of secondary cesarean section (11.1% versus 19.9%, *P* = 0.01). However, the rate of overall cesarean section was similar among the two groups, but higher with respect to the rate of cesarean section observed in the general GDM Italian population (34.9%) [33]. The lack of differences in proportions for adverse GDM-related events among these two groups of women with GDM further indicated the importance of identifying and treating all pregnants with GDM.

## 4. Discussion

In this paper, we investigated the effectiveness of the new Italian guidelines for the screening of GDM, according to which OGTT is not recommended in <35-year-old women without defined risk factors for GDM. We demonstrated that following these guidelines, over 30% of these women would miss GDM diagnosis. This is particularly relevant, since this group showed a significant risk for serious adverse maternal and neonatal hyperglycaemia-related events, including major rate of primary cesarean section, polyhydramnios, preterm delivery, admission to NICU, LGA, and higher neonatal weight, with regard to healthy, pregnant <35-year-old women without risk factors for GDM. In addition, the rate of adverse events in this group was similar to all the other women with GDM, further supporting the notion that—among pregnants <35 years old without risk factor for GDM—women with GDM must be identified and appropriately treated to reduce the rate of maternal and neonatal complications. Similar findings have been recently reported [34, 35].

The group of women with GDM were significantly older and had a significantly higher BMI compared to the healthy group. However, all the observed associations were maintained even after correction for age and prepregnancy BMI, indicating that GDM was an independent risk factor from age and maternal BMI. Concerning maternal BMI, previous observations demonstrated that GDM and increased BMI were independently associated with adverse maternal and neonatal outcomes, and that their combination had a greater impact than either one alone [36–39]. Our findings confirm and extend these observations, given that some adverse pregnancy outcomes were correlated with prepregnancy BMI, even within the normal range (<25 kg/m^2^), and independently from GDM.

Associations between maternal glucose levels and pregnancy outcomes were observed in all centers participating to the Hyperglycemia and Adverse Pregnancy Outcomes (HAPO) international study [11], thus justifying the development of global criteria for GDM [15]. However, lines of evidence—emerged from the worldwide debate over the adoption of the IADPSG recommendations—indicated that screening criteria must be modulated on the basis of population characteristics and public health available budget [19]. In this regard, our results show that most cases of GDM were diagnosed at baseline and at 1 hr of OGTT timeframe. In 4.7% of women, in which diagnosis occurred at 2 hr of OGTT timeframe, only one case of adverse outcome (polyhydramnios) was identified. Thus, consistently with similar evidences [19, 23], our findings suggest that in the Italian population, a 1 h OGTT might be justified for cost effectiveness reasons, and this strategy might increase patient adherence to the test, ensuring additional cases of GDM.

The main limitation of this study is that the sample size was not large enough to allow the detection of slight differences in maternal and neonatal outcomes (type 2 error). Also, the higher rates of preterm delivery observed among GDM cases, together with the increase of both cesarean section and the newborn admitted to NICU rates could induce the suspicion of excessive medical interventions. However, the major rate of polyhydramnios and LGA in GDM women explains the higher number of cesarean sections in this group, whereas the presumed overtreatment does not explain the neonatal primary outcomes, such as LGA, and the higher neonatal weight. Finally, it must be considered that all outcomes in our GDM groups are markedly lower with respect to those observed in a multicenter Italian study on GDM [33].

## 5. Conclusions

In summary, our study indicates that the new Italian recommendations are less effective with respect to the IADPSG criteria in preventing the adverse events linked to GDM. Although further investigations are required to corroborate our findings in other female populations, the choice of limiting OGTT to 1 hr, while extending the test to all pregnant women, could represent a better strategy in terms of cost effectiveness.

## Figures and Tables

**Table 1a:** (a)

Women	GDM (*N* = 171)	No GDM (*N* = 367)	*P* value
Race	Caucasian	Caucasian	—
Age (yr), median	32	30	<0.001
Mean ± SD	30.8 ± 3.2	29.3 ± 3.5	
BMI (Kg/m^2^), median	23.4	21.4	<0.001
Mean ± SD	22.8 ± 1.9	21.4 ± 2.0	
Gravidity (*n*), median	1	1	0.934
Mean ± SD	1.5 ± 0.7	1.5 ± 0.6	
Week of OGTT, median	27	27	0.230
Mean ± SD	27.1 ± 1.2	27.1 ± 0.8	
Glycaemia at 0' (mg/dL), median	92.0	80.0	<0.001
Mean ± SD	90.4 ± 8.57	79.6 ± 5.6	
Glycaemia at 60' (mg/dL), median	181.0	125.0	<0.001
Mean ± SD	171.6 ± 27.5	126.2 ± 25.3	
Glycaemia at 120' (mg/dL), median	130.0	100.0	<0.001
Mean ± SD	130.5 ± 22.8	101.7 ± 19.2	
Increasing weight at OGTT (Kg), median	10.0	7.0	<0.001
Mean ± SD	9.8 ± 3.4	7.0 ± 2.7	
Increasing weight at delivery (Kg), median	10.0	8.0	<0.001
Mean ± SD	10.3 ± 3.4	8.0 ± 2.8	

Comparisons among groups were performed by Mann-Whitney *U* test. SD: standard deviation; BMI: body mass index.

**Table 1b:** (b)

Trait	At OGTT time	At delivery	*P* value
HbA1c (%), median	5.2	5.1	0.001
Mean ± SD	5.2 ± 0.3	5.1 ± 0.3	
Range	4.2–6.1	4.0–5.9	
Increasing weight (Kg) median	10.0	10.0	0.131
Mean ± SD	9.8 ± 3.4	10.3 ± 3.4	

Comparison was performed by Mann-Whitney *U* test. SD: standard deviation.

**Table 2a:** (a)

Outcome	GDM (*N* = 171)	No GDM (*N* = 367)	OR (95% CI)	*P* value	OR (95% CI)^a^	*P* value^a^	Power (%)
Primary cesarean section, *N* (%)	51 (29.8)	56 (15.3)	2.36 (1.53–3.64)	<0.001	1.92 (1.21–3.06)	0.006	>95
Secondary cesarean section, *N* (%)	19 (11.1)	12 (3.7)	3.97 (1.80–8.77)	0.001	5.05 (2.11–12.08)	<0.001	85.3
Cesarean section after labor, *N* (%)	3 (1.8)	10 (2.7)	0.64 (0.17–2.35)	0.498	0.55 (0.14–2.21)	0.401	9.6
Labor induction, *N* (%)	2 (1.2)	1 (0.3)	4.33 (0.39–48.10)	0.233	3.85 (0.28–53.29)	0.314	13.6
Gestosis manifestations, *N* (%)	12 (7.0)	11 (3.0)	2.44 (1.05–5.65)	0.037	2.03 (0.83–4.97)	0.120	48.7
Hypertension, *N* (%)	7 (4.1)	6 (1.6)	2.57 (0.85–7.76)	0.095	1.73 (0.70–7.16)	0.173	33.5
Preeclampsia, *N* (%)	5 (2.9)	5 (1.4)	2.18 (0.62–7.63)	0.223	1.70 (0.44–6.65)	0.443	18.2
Fetal distress, *N* (%)	5 (2.9)	10 (2.7)	1.07 (0.36–3.20)	0.896	0.91 (0.28–3.00)	0.879	8
Polyhydramnios, *N* (%)	8 (4.7)	4 (1.1)	4.45 (1.32–15.0)	0.016	4.48 (1.20–16.73)	0.025	58.8
Oligohydramnios, *N* (%)	4 (2.4)	3 (0.8)	2.91 (0.64–13.13)	0.166	1.65 (0.32–8.51)	0.549	28.6
Preterm delivery, *N* (%)	14 (8.2)	13 (3.5)	2.43 (1.11–5.29)	0.025	1.94 (0.84–4.48)	0.118	52.4
Breech presentation, *N* (%)	18 (10.5)	31 (8.4)	1.23 (0.67–2.26)	0.502	1.24 (0.65–2.21)	0.563	9.9

OR: odds ratio; CI: confidence interval.

^a^Values were adjusted for maternal age, pregravidic BMI, and parity.

Power has been post hoc calculated with G*Power 3.1, entering *R*-squared multiple correlation coefficient obtained with regression for each trait.

**Table 2b:** (b)

Outcome	GDM (*N* = 171)	No GDM (*N* = 367)	OR (95% CI)	*P* value	OR (95% CI)	*P* value	Power (%)
Birth weight (Kg), mean ± SD (median)	3.2 ± 0.4 (3.3)	3.09 ± 0.3 (3.1)	—	0.002^a^	—	<0.001^b^	>95
Serious perinatal complications, *N* (%)	20 (11.7)	32 (8.7)	1.47 (0.82–2.63)	0.199	1.24 (0.66–2.33)^ c^	0.497^c^	17.3
Dystocia, *N* (%)	0 (0.0)	1 (0.3)	—	—	—	—	—
Bone fracture, *N* (%)	2 (1.2)	0 (0.0)	—	—	—	—	—
Admission to NICU, *N* (%)	11 (6.4)	6 (1.6)	4.14 (1.50–11.38)	0.006	4.39 (1.44–13.37)^d^	0.009^d^	68.6
RDS, *N* (%)	3 (1.8)	2 (0.5)	3.26 (0.54–19.69)	0.198	2.66 (0.41–17.44)^e^	0.307^e^	26.4
TTN, *N* (%)	4 (2.4)	3 (0.8)	2.91 (0.64–13.13)	0.166	1.89 (0.33–10.70)^e^	0.472^e^	27.9
Macrosomy (≥4 Kg), *N* (%)	2 (1.2)	6 (1.6)	1.44 (0.24–8.67)	0.693	0.48 (0.86–2.70)^c^	0.481^c^	28.6
LGA, *N* (%)	15 (8.8)	7 (1.9)	4.94 (1.98–12.37)	<0.001	3.53 (1.34–9.34)^c^	0.011^c^	85.6
SGA, *N* (%)	5 (2.9)	6 (1.6)	1.81 (0.54–6.02)	0.332	1.98 (0.53–7.41)^c^	0.312^c^	16.6
Metabolic complications, *N* (%)	9 (5.3)	7 (1.9)	2.86 (1.05–7.80)	0.041	2.32 (0.76–7.06)^c^	0.137^c^	46.6
Hypoglycaemia, *N* (%)	1 (0.6)	0 (0.0)	—	—	—	—	—
Hyperbilirubinemia, *N* (%)	4 (2.4)	3 (0.8)	2.91 (0.64–13.13)	0.166	1.19 (0.24–5.84)^c^	0.826^c^	28.5
Hypocalcemia, *N* (%)	2 (1.2)	2 (0.5)	2.16 (0.30–15.46)	0.443	5.28 (0.67–41.37)^c^	0.113^c^	15.4
Polycythemia, *N* (%)	2 (1.2)	2 (0.5)	2.16 (0.30–15.46)	0.443	2.19 (0.26–18.69)^c^	0.474^c^	15.4

^a^Calculated with Mann-Whitney test. ^b^Calculated by linear regression analysis after adjustment for maternal age, pregravidic BMI, and gestational age at birth. ^c^Values were obtained with logistic regression analysis after adjustment for maternal age, pregravidic BMI, parity, and gestational age at birth. ^d^Values were obtained with logistic regression analysis after adjustment for maternal age, pregravidic BMI, parity, and neonatal weight. ^e^Values were obtained with logistic regression after adjustment for maternal age, pregravidic BMI, parity, and mode of delivery. NICU: neonatal intensive care unit; RDS: respiratory distress syndrome; TTN: transient tachypnea of newborn; LGA: large for gestational age; SGA: small for gestational. Power has been post hoc calculated with G*Power 3.1, entering *R*-squared multiple correlation coefficient obtained with regression for each trait.

**Table 3a:** (a)

Outcome	<35 yr old no risk (*N* = 171)	≥35 yr old or with risk factor(s) (*N* = 503)	*P* value
Primary cesarean section, *N* (%)	51 (29.8)	80 (15.9)	<0.001
Secondary cesarean section, *N* (%)	19 (11.1)	100 (19.9)	0.010
Cesarean section after labor, *N* (%)	3 (1.8)	20 (4.0)	0.224
Total cesarean section, *N* (%)	73 (42.7)	200 (39.8)	0.528
Labor induction, *N* (%)	2 (1.2)	13 (2.6)	0.377
Hypertension, *N* (%)	7 (4.1)	19 (3.8)	0.821
Preeclampsia, *N* (%)	5 (2.9)	12 (2.4)	0.778
Fetal distress, *N* (%)	5 (2.9)	11 (2.2)	0.567
Polyhydramnios, *N* (%)	8 (4.7)	16 (3.2)	0.347
Oligohydramnios, *N* (%)	4 (2.4)	10 (2.0)	0.760
Preterm delivery, *N* (%)	14 (8.2)	27 (5.4)	0.196
Breech presentation, *N* (%)	18 (10.5)	25 (5.0)	0.017

2-tailed Fisher exact test was used for comparisons of proportions.

**Table 3b:** (b)

Outcome	<35 yr old no risk (*N* = 171)	≥35 yr old or with risk factor(s) (*N* = 503)	*P* value
Birth weight (Kg), mean ± SD	3.2 ± 0.4	3.2 ± 0.4	0.427*
Neonatal mortality, *N* (%)	0	1 (0.2)	>0.999
Dystocia, *N* (%)	0 (0.0)	1 (0.2)	>0.999
Bone fracture, *N* (%)	2 (1.2)	6 (1.2)	>0.999
Admission to NICU, *N* (%)	11 (6.4)	31 (6.2)	0.856
RDS, *N* (%)	3 (1.8)	9 (1.8)	>0.999
TTN, *N* (%)	4 (2.4)	11 (2.2)	>0.999
Macrosomy (≥4 Kg), *N* (%)	2 (1.2)	16 (3.1)	0.269
LGA, *N* (%)	15 (8.8)	43 (8.5)	>0.999
SGA, *N* (%)	5 (2.9)	14 (2.8)	>0.999
Metabolic complications, *N* (%)	9 (5.3)	30 (6.0)	0.851
Hypoglycaemia, *N* (%)	1 (0.6)	4 (0.8)	>0.999
Hyperbilirubinemia, *N* (%)	4 (2.4)	12 (2.4)	>0.999
Hypocalcemia, *N* (%)	2 (1.2)	8 (1.6)	>0.999
Polycythemia, *N* (%)	2 (1.2)	6 (1.2)	>0.999

*Comparison has been performed with the Mann-Whitney *U* test. 2-tailed Fisher exact test was used for comparisons of proportions. SD: standard deviation; NICU: neonatal intensive care unit. TTN: transient tachypnea of newborn; LGA: large for gestational age; SGA: small for gestational age; RDS: neonatal respiratory distress syndrome.
